# When, why and how are estimated effects transported between populations? A scoping review of studies applying transportability methods

**DOI:** 10.1007/s10654-025-01217-w

**Published:** 2025-04-18

**Authors:** Fabian Manke-Reimers, Vincent Brugger, Till Bärnighausen, Stefan Kohler

**Affiliations:** 1 Center for Preventive Medicine and Digital Health, Medical Faculty Mannheim, Heidelberg University, Röntgenstraße 7, 68167 Mannheim, Germany; 2 Heidelberg Institute of Global Health, Medical Faculty and University Hospital, Heidelberg University, Heidelberg, Germany; 3 Department of Global Health and Population, Harvard T.H. Chan School of Public Health, Boston, MA USA; 4 Africa Health Research Institute, Durban, South Africa; 5 Institute of Social Medicine, Epidemiology and Health Economics, Charité– Universitatsmedizin Berlin, Corporate Member of Freie Universitat Berlin and Humboldt-Universitat zu Berlin, Berlin, Germany

**Keywords:** Transportability, Generalizability, External validity, Scoping review, Causal inference

## Abstract

Transportability methods can improve the external validity of estimated effects by accounting for effect heterogeneity due to differently distributed covariates between populations. This scoping review aims to provide an overview of when, why and how transportability methods have been applied. We systematically searched MEDLINE (Ovid), Embase, Web of Science, EconLit and Google Scholar for studies published between 2010 and December 18, 2024. Studies using transportability methods in a numerical application for at least partly non-overlapping source and target populations were included. We identified 3432 unique studies and included 64 studies applying transportability methods. Over two thirds of the included studies (44/64) introduced new methods. Less than one third of the included studies (20/64) were pure applications of transportability methods. Most applied studies (17/20) transported effect estimates from randomized controlled trials. Effects were transported to target populations with either complete (9/20) or no (9/20) treatment and outcome data or both (2/20). The most frequent aims of applied studies were to transport estimated effects to new populations (10/20) and to assess effect heterogeneity explainable by measured covariates (8/20). How transportability methods were applied varied widely between studies, for instance in the covariate selection approach and sensitivity analysis. Methodological studies with a transportability application presented new transportability estimators for randomized data (5/44), specific transportability applications (e.g., meta-analysis, mediation analysis; 21/44) and other methodological aspects (e.g., covariate selection, missing data handling; 18/44). Transportability methods are a useful tool for knowledge transfer between populations. More applications of transportability methods and guidance for their use are desirable.

**Supplementary Information:**

The online version contains supplementary material available at 10.1007/s10654-025-01217-w.

## Introduction

The external validity of an effect estimate is of interest when aiming to infer from a study to a population that is not (a random sample of) the study sample [1, 2]. Achieving external validity is difficult for many reasons and across study types. The effect of a treatment may differ between populations due to differently distributed covariates [3–5]. A randomized controlled trial (RCT) could be internally valid and result in unbiased effect estimates for the study sample. Yet, the RCT might examine a non-random sample of the target population or a population distinct from the target population and hence lack external validity [1, 4, 6–8]. Observational studies may aim to represent a population of interest, but their results might not apply to other target populations [9–11]. Meta-analyses or multicenter RCTs can generate pooled effect estimates but leave unclear how the pooled estimates apply to a specific population [12–14].

Repeating a study in a random sample of a target population is an option to obtain valid effect estimates in a new population. However, infrastructure, money, time, and ethical considerations may prevent repeating a study or make it unattractive. Transportability methods are a relatively novel approach to address effect heterogeneity between populations. They aim to improve the external validity of study results by transporting effect estimates from a source population to a target population by adjusting for differently distributed covariates. Transportability methods might be applied in settings in which the source population and the target population are (at least partly) non-overlapping [2, 15–17]. Previous reviews summarized transportability assumptions, transportability estimators and data constellations under which effect estimates might be transported [16, 18, 19]. In contrast to our study, previous reviews conducted no systematic literature search and focused on transportability methods rather than on their applications.

We conducted a scoping review of studies applying transportability methods. For applied studies of transportability methods, we provide an overview of when, why and how effect estimates were transported. For methodological studies with a transportability application, we provide an overview of the presented methods and types of applications.

## Methods

We conducted a scoping review of studies applying transportability methods, following the **P**referred **R**eporting **I**tems for **S**ystematic reviews and **M**eta-**A**nalyses extension for **Sc**oping **R**eviews (PRISMA-ScR) Checklist [20] (Supplement A).

### Information sources and search strategy

We last searched MEDLINE (Ovid), Embase, Web of Science, EconLit and Google Scholar on December 18, 2024. We combined the search terms “transportability” or “transport” with search terms referring to a research study and excluded the term “transporter” and variations thereof. Title, abstracts and, where available, keywords and full texts were searched. The database searches were restricted to studies published in 2010 or later, as transportability began to emerge in the literature in the early 2010s [7, 16, 18, 21]. The complete search syntax used in each database is provided in the supplementary material (Supplement B).

### Study screening

After removing duplicates from the search results, two authors (FMR and VB) independently screened the titles and abstracts of the identified studies. The same two authors independently screened the full texts of studies selected for eligibility. Conflicts were resolved by discussion until consensus was reached. The study screening was performed using the Covidence software [22].

### Inclusion and exclusion criteria

We included studies in the scoping review that, firstly, transported an estimated effect from a source population to a target population in a numerical application and, secondly, addressed a transportability problem. We considered the latter as fulfilled if a study was conducted in a setting in which at least some members of the source population sample have a 0% probability of being in the sample from the target population (a definition frequently used in the literature [2, 15, 23, 24]). No exclusion criteria were applied.

### Categorization of studies

We distinguished between studies exclusively applying transportability methods (referred to as applied studies) and studies with an application but a focus on developing methods in relation to transportability (referred to as methodological studies). Applied studies aimed to address a practical research question by applying an existing transportability method. Methodological studies aimed to develop methods and included a transportability application of the new method. Within methodological studies, we further distinguished whether studies presented new transportability estimators for randomized data, specific transportability applications (e.g., meta-analysis, mediation analysis) or other methodological aspects (e.g., covariate selection, missing data handling). Studies were independently categorized by two authors (FMR and VB) and conflicts were resolved by discussion.

### Data extraction

Data was extracted by one author and cross-checked by a second author (FMR and VB). We extracted the authors, publication year and the data source(s) for all studies and specific data for applied studies and methodological studies.

### Applied studies of transportability methods

For applied studies, we extracted the source population and target population data (e.g., RCT, observational study, multicenter trial) and whether the target population data contained treatment (or exposure of interest data for observational studies) and outcome data (**“When?”**). We further extracted the study topic and the reason for using transportability methods (**“Why?”**) . To characterize how transportability methods were applied, we extracted which aspects of the transportability method application were reported and what was reported. We expanded the extracted aspects iteratively based on the full text assessment (**“How?”**) .

### Methodological studies with a transportability application

For methodological studies with a transportability application, we extracted the introduced method and assumptions related to the method. We further extracted which type of transportability application was provided (e.g., real-world example, simulation, toy example). When studies presented new estimators to transport estimates from randomized data to other data, we extracted the stated robustness of the estimator. When studies presented specific transportability applications, we extracted which application was addressed. When other methodological aspects were studied, we extracted which problem was addressed.

## Results

**Fig. 1:**
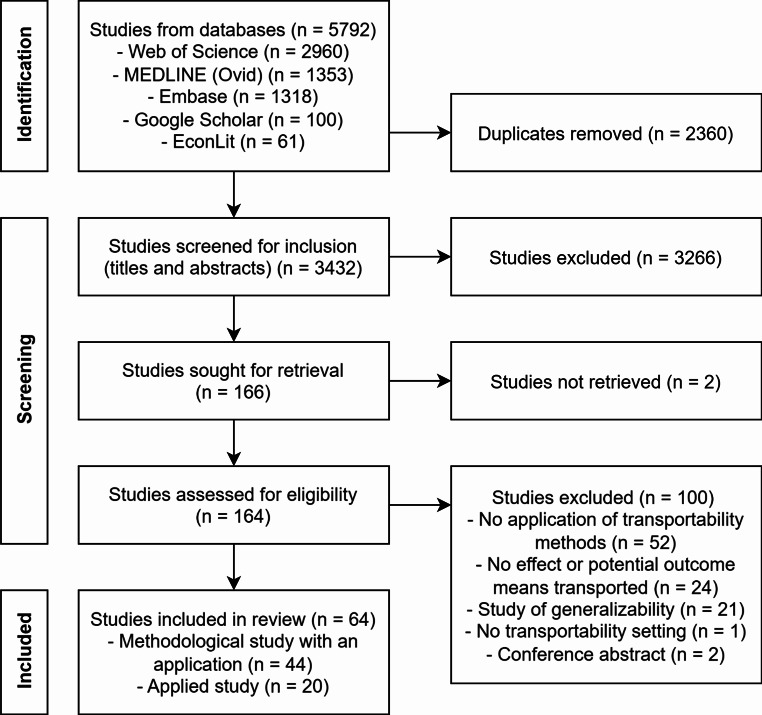
Flow chart of studies with transportability method applications that were identified, screened and included in the scoping review

Our database search identified 5792 studies. After removing 2360 duplicates, we screened the title and abstract of 3432 studies. The full texts of 166 studies were retrieved and assessed for eligibility. The inclusion criteria were met by 64 studies with transportability applications. Among these, less than one third of the studies with transportability applications were applied studies (20/64) [12, 17, 25–42]. Over two thirds were studies with a focus on introducing new methods (44/64) [1, 9, 13, 14, 18, 23, 24, 43–79] (Fig. 1).

**Fig. 2:**
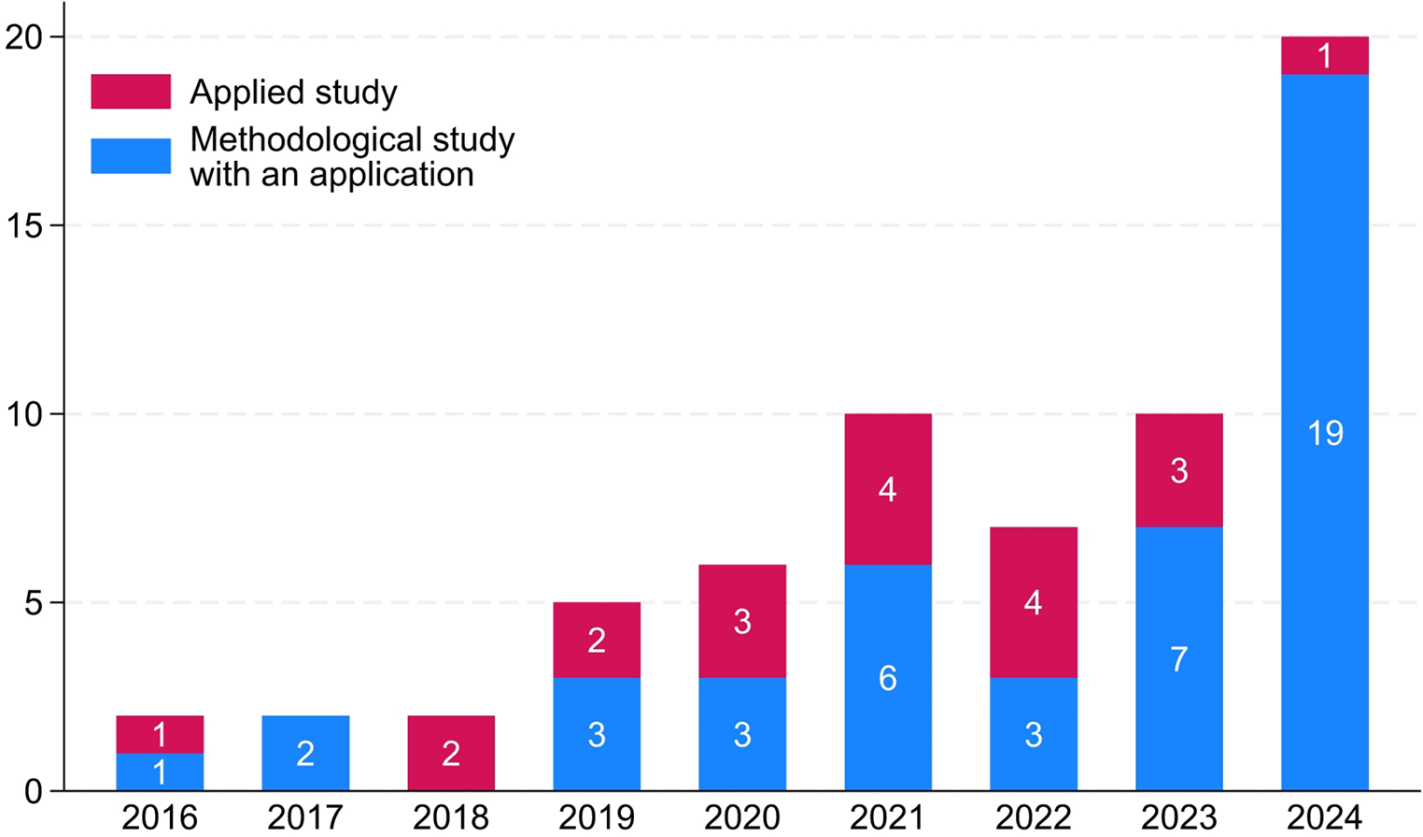
Number of studies applying transportability methods by publication year and study type. The systematic literature search included studies published between January 1, 2010, and December 18, 2024

The number of studies with transportability applications published each year rose substantially on a low level from the first published studies in 2016 (2/64) to December 18, 2024 (20/64). Almost half (30/64) of all studies with transportability applications were published in the two years preceding our literature search. In 2024, the year with the highest number of published studies with a transportability application (20/64), fewer applied studies of transportability methods were published (1/20) than in any other year since 2017 (Fig. 2).

### When estimated effects were transported in applied studies

Most of the applied studies used source population data from multicenter RCTs (16/20) [12, 17, 26–33, 35, 37–41]. Other studies used source population data from an observational study (3/20) [25, 34, 36] or from several RCTs (1/20) [42]. Data sources on target populations were most often observational data (11/20) [25, 26, 28–30, 32–34, 36, 40, 41] followed by data from RCTs (6/20) [12, 27, 30, 37, 38, 42]. Some authors used hypothetical target populations (3/20) [17, 31, 35] or simulated data based on observational data due to data use restrictions (1/20) [39]. Treatment and outcome data on the target population were as often completely available (to which we refer to as complete data) for the target population (9/20) [25, 27, 34, 36–40, 42] as unavailable (9/20) [17, 26, 28, 29, 31–33, 35, 41]. In some studies (2/20), treatment and outcome data in the target population were partially available, for instance, when different treatments were compared and data were pooled from different sources (1/20) [12] or when different target populations were assessed (1/20) [35] (Table 1).

The most frequent study setting in which transportability methods were applied was transporting an effect from a multicenter RCT to a target population with observational or hypothetical data without complete data (10/20) [17, 26, 28–33, 35, 41]. Transporting an effect from a source population with RCT data to a target population with complete RCT data was the second most frequent study setting (6/20) [12, 17, 27, 37, 38, 42]. Transporting an effect from a source population with observational data to a target population with observational data was conducted by three studies [25, 34, 36]. Two studies with complete data on both the source and target populations transported an effect from a source population with RCT data to a target population based on observational data [39, 40]. Two further studies used a meta-analysis to estimate an effect for a target population with data from distinct RCTs on the source population and complete RCT data on the target population [12, 42].

### Why estimated effects were transported in applied studies

#### Studies without complete target population data

One reason for using transportability methods without complete data on the target population was that studies aimed to infer from effects that were estimated in RCTs to larger populations (7/10) [26, 28–30, 32, 33, 41] or to hypothetical data which might represent specific target populations (3/10) [17, 31, 35]. One study compared different treatments in the target population using RCT data as the source and observational data on the target population [30] (Table 1).

**Table 1:** Applied studies of transportability methods: why and when estimated effects were transported between populations

Authors	Year	Study topic	Reason for using transportability methods (“Why?”)	Source population data (“When?”)	Target population data (“When?”)	Complete target population (“When?”)	Data source(s)
Barker et al. [12]	2022	Challenges of causal meta-analysis, effect of emotion regulation, family processes and HIV-related knowledge and skills on HIV prevention	Estimating potential outcome means for each treatment and average treatment effects for comparing treatments in each target population	RCTs (M), partially with differing treatments	RCTs (M)	Partially	STAR trial, Therapeutic schools: affect management and HIV prevention, Project STYLE
Basu et al. [25]	2021	Determining whether and why use of fruit and vegetable vouchers are associated with varied nutritional intake across different populations and environments	Studying whether the differences in voucher outcomes between study sites could be associated with measured differences between the groups or with unmeasured confounding	Observational	Observational	Yes	Own study data
Bengtson et al. [26]	2016	Transporting the 6-month effect of the Measurement-Based Care (MBC) intervention on depression from the SLAM DUNC trial to a population of HIV-infected, depressed adults in routine care in the United States	Assessing what effect the intervention might have among HIV-infected depressed adults in routine care in a multisite observational cohort	RCTs (M)	Observational	No	SLAM DUNC trial, CNICS cohort
Berkowitz et al. [27]	2019	Effect of spironolactone and intensive blood-pressure treatment on cardiovascular disease	Determining whether transportability methods could suggest site anomalies not readily identified through standard methods	RCTs (M)	RCTs (M)	Yes	TOPCAT trial, ACCORD BP trial
Berkowitz et al. [28]	2018	Generalizing the largest clinical trial of intensive blood pressure treatment among adults with diabetes to the U.S. population	Transporting the results of multicenter trials to a more representative population of Americans with diabetes	RCTs (M)	Observational	No	ACCORD BP trial, NHANES survey
Hutcheon & Liauw [29]	2023	Estimating the real-world absolute risk reduction and number-needed to-treat for antenatal corticosteroids at late preterm ages, accounting for gestational age differences between the ALPS and real-world populations	Aiming to improve transportability by extending the ALPS findings to a population in which clinical guidelines informed by the trial are applied	RCTs (M)	Observational	No	ALPS trial, population-based cohort from British Columbia
Inoue et al. [30]	2023	Effect of individualized treatment effect of intensive systolic blood pressure control on the reduction in cardiovascular. Outcomes at three years. Comparing the performance of high-benefit approach versus the high-risk approach	Estimating and comparing high-benefit and high-risk approaches on systolic blood pressure in a survey sample of US adults	RCTs (M)	Observational	No	SPRINT trial, ACCORD-BP trial, NHANES survey
Inoue et al. [31]	2022	Investigating whether the association of intensive blood pressure control with cardiovascular events varies by living arrangement status among black and non-black individuals	Quantifying the potential benefits of intensive blood pressure control in external populations with different distributions of baseline characteristics to improve the generalizability of the trial findings	RCTs (M)	Hypothetical with varying distributions of covariates	No	SPRINT trial
Inoue et al. [17]	2021	Effect of screening on lung cancer mortality	Extrapolate findings from lung cancer screening trials to different target populations within the multicenter structure and hypothetical external populations	RCTs (M)	RCTs (M), hypothetical	Partially	NLST trial
Lund et al. [32]	2020	Comparing 5-year mortality in patients of two adjuvant chemotherapy regimens for colon cancer	Assessing effectiveness measured in an RCT to a more general population	RCTs (M)	Observational	No	MOSAIC trial, SEER-Medicare
Mollan et al. [33]	2021	Assessing whether population differences might explain the divergence of trial results on the effect of efavirenz for treatment of HIV	Evaluating what the effect of initiating efavirenz on suicidal thoughts/behaviors might have been had the trials been conducted in the target population	RCTs (M)	Observational	No	Four ACTG trials, CNICS cohort
Neophytou et al. [34]	2024	Assessing the presence of effect modification and transportability of effect estimates for secondhand smoke on birthweight between different cohorts	Assessing effect heterogeneity caused by measured participant-level sociodemographic characteristics and to which extend these characteristics can explain effect heterogeneity	Observational	Observational	Yes	ECHO cohorts
Prosper et al. [35]	2021	Effect of screening on lung cancer mortality	Evaluating the potential benefit of lung cancer screening among populations with different distributions of sex, smoking status and ethnicity	RCTs (M)	Hypothetical with varying distributions of covariates	No	NLST trial
Ramagopalan et al. [36]	2022	Evaluating whether the overall survival estimates for a selected group of patients from the US are transportable to Canadian patients	Assessing whether adjustments for pretreatment characteristics could reliably and robustly approximate overall survival from US patients to Canadian patients	Observational (M)	Observational (M)	Yes	Flatiron Health database, Province of Alberta, Alberta Cancer Registry
Rudolph et al. [37]	2020	Effect of a housing voucher on mental health or substance abuse disorder, understanding mediation mechanisms and the degree to which they are transportable across trial sites	Assessing the extent to which mediators contribute to site differences in indirect effect estimates. Comparing transported estimates to observed estimates	RCTs (M)	RCTs (M)	Yes	MTO trial
Rudolph et al. [38]	2018	Effect of a housing voucher on mental health or substance abuse disorder, testing the responsibility of covariates for differences in site effects	Assessing the extent to which differences in distributions of potential effect modifiers account for differences in effects across sites	RCTs (M)	RCTs (M)	Yes	MTO trial
Webster-Clark et al. [39]	2020	Compare two-year risk differences for ischemic stroke, mortality, and gastrointestinal bleeding in older adults with atrial fibrillation initiating dabigatran and warfarin	Comparing transported estimates to estimates with non-experimental methods	RCTs (M)	Simulated based on observational data due to data use restrictions	Yes	RE-LY trial, Medicaid
Webster-Clark et al. [40]	2019	Assessing the transportability of treatment effects from the standard of care for metastatic colorectal cancer control arm in a phase III trial (HORIZON III) to a target population	Evaluating assumptions for external validity using data from a phase III trial of chemotherapy for metastatic colorectal cancer by comparing all-cause mortality in the trial control arm (representing the current standard of care) to that of similarly treated patients in routine care	RCTs (M)	Observational	Yes	HORIZON III trial, Medicaid
Wei et al. [41]	2023	Transporting treatment effects from two heart failure trials to a heart failure registry in Scotland	Showing that heart failure trials can be calibrated to the more complex populations encountered in clinical practice with only moderate loss in precision	RCTs (M)	Observational	No	COMET trial, DIG trial, Scottish heart failure registry
Zuo et al. [42]	2022	Assessing differences of colon cancer treatment in adjuvant clinical trials	Investigating the reasons for effect heterogeneity across different trials with causal meta-analysis methods and comparing the results with the standard meta-analysis approach	RCTs	RCTs	Yes	ACCENT trial database

RCT = randomized controlled trial, M = multicenter trial

#### Studies with complete target population data

Studies applying transportability methods to infer from RCT data to complete RCT data aimed to examine how much effect heterogeneity is explainable by measured effect modifiers [17, 27, 38, 42] (4/11) or mediators [37] (1/11). Studies applying transportability methods to infer from observational data to complete observational data aimed to assess effect heterogeneity explained by measured effect modifiers or confounders (2/11) [25, 34] and how well effect estimates from a target population were approximated with transported effect estimates (1/11) [36]. Studies also compared estimated effects derived under different assumptions (1/11) [39] or calculated potential outcome means to assess to which extend trial results hold in a target population of interest (1/11) [40] when inferred from RCT to observational data. A study using pooled estimates for different treatments from a meta-analysis aimed to compare effects across treatments and target populations (1/11) [12]. Another study involving meta-analysis aimed to estimate an effect from multiple sources for the target population and to compare the result with estimated effects from standard meta-analysis (1/11) [42].

### How estimated effects were transported in applied studies

When describing how transportability methods were applied, studies frequently reported a population description, covariate selection approach, missing data handling, assessment of positivity and outcome model assumptions, transportability estimator, estimation model, and/or sensitivity analysis. Studies with treatment and outcome data on the target population further commonly reported if and how effect heterogeneity between populations was assessed (Table 2).

**Table 2:** Applied studies of transportability methods: how estimated effects were transported between populations

Authors	Year	Population description (“How?”)	Covariate selection approach (“How?”)	Missing data handling (“How?”)	Positivity and common outcome model assumption (“How?”)	Transportability estimator (“How?”)	Estimation model (“How?”)	Sensitivity analysis (“How?”)	Effect heterogeneity assessment (“How?”)
Barker et al. [12]	2022	Table with covariates and SMD	Identified by conditional random forest approach	Single imputation, predictive mean matching with chained equations	Discussed both	Outcome modelling, causal meta-analysis	Parametric	–	–
Basu et al. [25]	2021	Table with covariates and tests on MD	Measured potential effect modifiers	No missing covariates, missing outcome data not imputed	–	Entropy balancing	Nonparametric	Used different combinations of covariates	Calculated the overlap of confidence intervals for different covariate combinations
Bengtson et al. [26]	2016	Figures with covariate proportions or medians	Shifted effect measure modifiers	Multiple imputation, chained equations	Discussed common outcome model	IOSW	Parametric	Excluded patients based on rare characteristics or geography	–
Berkowitz et al. [27]	2019	Table with covariates and tests on MD	Based on prior literature and selection diagram, published DAG	Multiple imputation, random forest approach	Discussed common outcome model	TMLE	Parametric	Robustness checks with additional covariates	Used calibration metrics to evaluate whether expected values matched observed values in the target population
Berkowitz et al. [28]	2018	Table with covariates and SMD	Based on prior literature and own hypotheses	No imputation as missingness was < 5% for all variables	–	IOSW, TMLE	Parametric, Nonparametric	Non-parametric model specification, different estimators, other covariate combinations	–
Hutcheon & Liauw [29]	2023	–	Based on prior literature, only covariate is gestational age	–	Discussed common outcome model	IOSW	Parametric	–	–
Inoue et al. [30]	2023	Table with covariates	Included all baseline covariates	Random forest approach	–	IOSW	Nonparametric	Transportability analysis is framed as sensitivity analysis	–
Inoue et al. [31]	2022	Table with covariates	Adjustment for sociodemographic, lifestyle, clinical and laboratory covariates, published DAG	Full record analysis, < 0.3% with missing information	Discussed positivity	IOSW	Parametric	–	–
Inoue et al. [17]	2021	Table with covariates and tests on MD	Preintervention demographic characteristics	–	Discussed both	IOSW	Parametric	–	–
Lund et al. [32]	2020	Table with covariates	Potential effect modifiers	–	–	IOSW	Parametric	–	–
Mollan et al. [33]	2021	Table with covariates	Baseline participant characteristics	Predictive mean matching, random forest, full record analysis	Discussed common outcome model	IOSW	Parametric	Different imputation approaches	–
Neophytou et al. [34]	2024	Table with covariates	Potential confounders (or proxies of such), potential effect modifiers	Multiple imputation by chained equations	Discussed both, omnibus test for common outcome model	TMLE	Nonparametric	Assessing the impact of exposure misclassification	Calculated margins between estimated effects in source and target populations and the transported effect estimate
Prosper et al. [35]	2021	Table with covariates	Adjustment for race, sex and smoking status	–	–	IOSW	Parametric	–	–
Ramagopalan et al. [36]	2022	Table with covariates and SMD	Baseline covariates	Full record analysis, multiple and single imputation	Discussed common outcome model	Outcome modelling	Parametric	Different imputation approaches, changes in risk factor distributions	–
Rudolph et al. [37]	2020	Table with covariates	Based on causal assumptions of DAG, published DAG	Imputation with chained equations, 30 datasets	Omnibus test for common outcome model	TMLE	Parametric	–	Calculated margins between estimated effects in source and target populations and the transported effect estimate
Rudolph et al. [38]	2018	Table with covariates	Baseline covariates	Imputation with chained equations, 30 datasets	Discussed both, omnibus test for common outcome model	TMLE	Nonparametric	–	Calculated margins between estimated effects in source and target population and the transported effect estimate
Webster-Clark et al. [39]	2020	Table with covariates and SMD	From trial subgroup analyses and clinical knowledge, published DAG	No missing data	Discussed both	IOSW	Parametric	Sampling model changes and additional target populations	–
Webster-Clark et al. [40]	2019	Table with covariates and SMD	Potential effect modifiers based on prior literature and expert opinion	Complete case analysis	–	IOSW	Parametric	Different covariate sets, impact of censoring weights, truncation of 95% IOSW percentile	–
Wei et al. [41]	2023	Table with covariates	All potential effect modifiers	Predictive mean matching, one dataset	Discussed positivity	Outcome modelling, IOSW	Parametric	Different estimators	–
Zuo et al. [42]	2022	Table with covariates	Common covariates collected across trials	Full record analysis	Discuss common outcome model	Outcome modelling, causal meta-analysis	Parametric	Used multiple survival models	Calculated prediction errors of models using transportability methods

SMD = standardized mean difference, MD = mean difference, DAG = directed acyclic graph, IOSW = inverse odds of sampling weights, TMLE = targeted maximum likelihood estimation

#### Population description

Most studies summarized the source and target population and their differences in a table (18/20) [12, 17, 25, 27, 28, 30–42]. Some studies additionally calculated the standardized mean differences of measured potential covariates between the populations (5/20) [12, 28, 36, 39, 40] or applied a test for mean differences (3/20) [17, 25, 27].

#### Covariate selection approach

Some studies appeared to use prior literature, clinical knowledge or expert opinion to support the covariate selection in their application of transportability methods (4/20) [27, 29, 39, 40]. Another approach was to include measured covariates that differed in their distributions between the populations (1/20) [26]. One study used a random forest approach to select the covariates data-driven (1/20) [12]. A few studies presented directed acyclic graphs to support the covariate selection or to state the causal assumptions (4/20) [27, 31, 37, 39]. More than half of the included applied studies did not report a specific approach to covariate selection and stated to include measured potential effect modifiers, covariates or baseline characteristics in the analysis (12/20) [17, 25, 30–36, 38, 41, 42].

#### Missing data handling

Studies commonly (16/20) described how they handled missing data. About one third of the studies (6/20) conducted listwise deletion and excluded the entire record from analysis if any single value was missing [28, 31, 33, 36, 40, 42]. Half of the studies (10/20) described using single imputation, multiple imputation, random forest, or predictive mean matching to replace missing data [12, 26, 27, 30, 33, 34, 36–38, 41].

#### Positivity and common outcome model assumptions

The assumptions of positivity and a common outcome model across the source and target population were assessed by all but six studies (14/20). Studies discussed the assumption of a common outcome model (7/20) [26, 27, 29, 33, 36, 37, 42], the positivity assumption (2/20) [31, 41] or both assumptions (5/20) [12, 17, 34, 38, 39]. Three studies tested for a common outcome model which is only possible with complete data on the target population [34, 37, 38].

#### Transportability estimator

The most frequently used estimator to transport effects was the inverse odds of sampling weights (12/20) [17, 26, 28–33, 35, 39–41]. Outcome modelling (4/20) [12, 36, 41, 42] and doubly robust estimators like targeted maximum likelihood estimation (5/20) [27, 28, 34, 37, 38] and entropy balancing (1/20) [25] were also used. Some studies used outcome modelling within a meta-analysis (2/20) [12, 42].

#### Estimation model

Most studies used a parametric estimation model for the transportability estimator (17/20) [12, 17, 26–29, 31–33, 35–37, 39–42]. A few studies used non-parametric models (3/20) [25, 28, 34, 38].

#### Sensitivity analysis

More than half of the studies reported conducting a sensitivity analysis (12/20). Two studies varied the approaches to missing data imputation [33, 36]. Two studies excluded patients based on their characteristics [26, 40]. Four studies varied the covariate set [25, 27, 28] or the covariate distributions [36]. Three studies analyzed multiple target populations [17, 31, 40]. One study changed from a parametric to a nonparametric model in the sensitivity analysis [28]. Other studies estimated transported effects with different estimators [28, 41], variations of parametric models [39, 42] or truncated the 95% percentile of weights for the analysis [40]. One study assessed the impact of exposure misclassification [34] and another study framed the transportability analysis itself as a sensitivity analysis [30].

#### Effect heterogeneity assessment

More than half of the studies with complete data on the target population assessed the effect heterogeneity between the transported effect estimate and the effect estimate from the target population (6/11) [25, 27, 34, 37, 38, 42]. Three studies calculated the margin between the estimated effect in the source population and the transported effect estimate divided by the margin between the estimated effect in the source and target populations. The resulting proportion was then interpreted as the effect heterogeneity explained by the included covariates [34, 37, 38]. Other studies used calibration metrics to evaluate whether expected values matched observed values in the target population (1/11) [27] or interpreted the overlap of confidence intervals for specific covariate combinations to assess their importance (1/11) [25]. Another approach for comparing effect estimates was to calculate the prediction errors of models using transportability methods (1/11) [42]. No study without complete data on the target population compared the transported estimate to an effect estimate from the target population.

### Overview of methodological studies with a transportability application

Methodological studies presented estimators to transport estimates from randomized data to other data (5/44) [23, 24, 43–45], specific transportability applications (21/44) [9, 13, 14, 46–63] and other methodological aspects in relation to transportability (18/44) [1, 18, 64–79] (Table 3).

**Table 3:** Overview of methodological studies with a transportability application

Authors	Year	Method introduced	Robustness or problem addressed	Assumptions related to method	Type of transportability application	Data source(s)
**Studies of new estimators to transport estimates from randomized data to other data**						
Dahabreh et al. [23]	2020	Augmented inverse weighting	Sampling or outcome model correctly specified	Exchangeability, positivity	Simulation, real-world example	CASS trial
Goldstein et al. [43]	2019	Outcome model approach	Outcome model correctly specified	Not stated	Real-word example	NAVIGATOR trial, Duke University Health System
Josey et al. [44]	2021	Entropy balancing (calibration weighting estimator)	Either linear conditional log odds hold or conditional linearity	Exchangeability, positivity, conditional linearity of potential outcomes, linear conditional log-odds of trial participation	Simulation, real-world example	ACCORD-BP trial; NHANES
Rudolph & van der Laan [45]	2017	Targeted maximum likelihood estimation	Sampling or outcome model correctly specified (in mediation analysis also the mediating model)	Exchangeability, positivity, common outcome model	Simulation, real-world example	MTO trial
Westreich et al. [24]	2017	Inverse odds of sampling weights	Sampling model correctly specified	Exchangeability, positivity	Toy example	–
**Studies of specific transportability applications**						
Bonander et al. [46]	2021	Linking auxiliary register data to participants and non-participants of studies	Transportability can be used with register data to debias the selection of participants	Exchangeability, positivity	Real-world example	SCAPIS cohort study, LISA database
Cao et al. [47]	2024	Transportability of counterfactual survival functions	Introducing estimators for transporting survival functions from randomized data to a complex survey	Exchangeability, positivity, covariate-dependent censoring	Simulation, real-world example	ACCORD-BP trial, NHANES
Dahabreh et al. [48]	2024	Extending identification results for transportability to relative effect measures	Proposing conditions and estimators for transportability with relative effect measures	Exchangeability, positivity	Real-world example	CASS trial
Dahabreh et al. [49]	2024	Benchmarking effect sizes	Providing a framework for benchmarking and synthesizing target trials from experiments and observational studies	Exchangeability, positivity	Real-world example	TASTE trial, SWEDEHEART registry
Dahabreh et al. [13]	2023	Refining assumptions of causally interpretable meta-analysis	Providing doubly robust estimators and corresponding assumptions for causal meta-analysis	Exchangeability, positivity	Real-world example	HALT-C trial
Dahabreh et al. [14]	2020	Causally interpretable meta-analysis	Giving meta-analytic results valid causal interpretation outside the trials included by using a defined target population	Exchangeability, positivity	Simulation, real-world example	HALT-C trial
Elliott et al. [50]	2023	Extending transportability methods to non-probability samples	Suggesting an estimator to deal with the target sample not being representative for the target population	Exchangeability, positivity	Simulation, real-world example	PAC-Man trial
Hayes-Larson et al. [51]	2024	Observational transportability	Presenting identifiability conditions and estimators for observational transportability	Exchangeability, positivity	Simulation	–
Josey et al. [9]	2022	Methods for transporting observational results	Extending transportability estimators for experimental data to also account for confounding bias	Exchangeability, positivity, conditional linearity of potential outcomes, conditional linear log-odds for sampling and treatment	Simulation, real-world example	United States Veterans Affairs (VA)
Kabali & Ghazipura [52]	2016	Computation of valid estimates of indirect treatment effects in network meta-analysis	Accounting for differing post-treatment variables in meta-regression	Exchangeability, positivity	Real-world example	Data from network meta-analysis for treatments for Bell’s palsy
Lee et al. [53]	2024	Transporting survival outcomes	Extending the augmented calibration weighting method to not require the proportional hazard assumption	Exchangeability, positivity	Simulation, real-world example	AIEDRP cohort study, HIV patient samples from Ethiopia and Thailand, ACTG 175trial
Lu et al. [54]	2023	Adjust site-level estimates in multicenter trials for differences in the distribution of observed unit-level features	Using transportability to address differences in trial site treatment effects which might be due to individual implementation at the center level or participant characteristics that vary between sites	Exchangeability, positivity	Real-word example	Project GAIN, Project Independence, National Evaluation of Welfare-to-Work Strategies (NEWWS)
Mehrotra et al. [55]	2021	Using transportability to make valid subgroup analyses in RCTs	Address differences in distribution of effect modifying covariates that bias effect estimates for any subgroup analyses that are made to take decisions on implementations of trials	Exchangeability, positivity	Real-world example	iPrEx study, Latino MSM Community Involvement Study
Rott et al. [56]	2024	Causal meta-analysis	Proposing a method to perform causally interpretable meta-analysis using aggregate and individual patient data	Exchangeability, positivity	Simulation, real-world example	Real-time Continuous Glucose Monitoring Data
Rudolph & Diaz [57]	2022	Incorporating multiple, high-dimensional mediators into transportability	Incorporating continuous mediators into transportability analysis as well as non-binary treatments	Exchangeability, positivity, no unmeasured confounding between mediator and outcome	Simulation and real-world example	MTO trial
Rudolph et al. [58]	2021	Transportability of mediation effects	Providing transportability estimators for analysis of mediated effects	Positivity, common outcome model	Simulation and real-world example	MTO trial
Shook-Sa et al. [59]	2024	Bridged treatment comparison	Introducing single-span estimators for treatment comparison without shared treatment arm	Exchangeability, positivity, no measurement error	Simulation, real-world example	ACTG 320 trial, ACTG 388 trial
Siddique et al. [60]	2024	Mixed data meta-analysis	Using transportability methods in meta-analysis with multiple treatments to estimate effects for studies missing specific treatment data	Exchangeability, positivity, data missing at random	Simulation, real-world example	64 observational studies consisting of 31 individual patient data and 33 aggregated data only studies
Van Lancker et al. [61]	2021	Transportability with near treatment positivity violations	Using transportability to estimate treatment effects in flexible treatment trials	Exchangeability, positivity	Simulation and real-world example	Study program on fixed and flexible dosing of chemotherapy
Vo et al. [62]	2019	Disentangling heterogeneity due to case-mix from other reasons	Providing valid aggregation of effects that were obtained from dissimilar source populations in meta-analysis	Exchangeability, positivity	Simulation and real-world example	IDP meta-analysis on vitamin D supplementation
Zivich et al. [63]	2024	Bridged treatment comparison	Compare treatment effects of RCTs with different comparison groups in a specific target population	Exchangeability, positivity	Real-world example	ACTG 175 trial, ACTG 320 trial
**Studies of other methodological aspects in relation to transportability**						
Barker et al. [65]	2023	Identifiability conditions for transportability with systematically missing data	Addressing heterogeneity among trials in causal meta-analysis due to systematic missing data	Exchangeability, positivity, conditions for systematic missing data	Simulations	–
Cinelli & Pearl [66]	2021	Using probabilities of causation for transportability to relax the transportability assumption	Relaxing the transportability assumption requires all mechanisms to match in the source and study population, estimating the effect when only some of those are the same	Positivity, independence of variables affecting the outcome, functional constraints on how these factors interact on the outcome	Simulation, real-world example	Two trials from Indonesia and Nepal on vitamin A supplementation on childhood mortality
Colnet et al. [18]	2024	Review on transportability and generalizability methods	Reviewing methods for causal inference on combining RCT and observational data	Not applicable for review article	Simulation, real-world example	CRASH-3 trial, Traumabase registry
Colnet et al. [67]	2024	Establishing expressions of bias and variance	Bounding bias, variance and quadratic risk as well as covariate selection for re-weighting procedures with categorical covariates	Exchangeability, positivity	Simulation, real-world example	CRASH-3 trial, Traumabase registry
Colnet et al. [68]	2022	Sensitivity analysis to account for missing covariates in transportability	Estimating the sensitivity of transportability to missing covariates	Exchangeability, positivity, covariates in target population identically distributed	Simulation, real-world example	CRASH-3 trial, Traumabase registry
Dahabreh et al. [69]	2023	Sensitivity analysis method without details on background knowledge	Addressing sensitivity analysis without relying on background knowledge and without observing a full set of covariates	Exchangeability, positivity	Real-world example	ACTG 175 trial, WIHS cohort study
Lund et al. [70]	2024	Visualizations to facilitate covariate selection	Presenting a practical roadmap and visualizations to assist extending study results to clinical practice settings and to assess model specification and performance	Positivity, exchangeability, correct model specification	Real-world example	MOSAIC trial, iKnowMed
Mayer et al. [71]	2023	Multiple imputation to handle missing data for transportability methods	Handling missingness in covariate data in transportability applications	Exchangeability, positivity, MCAR or MAR	Simulation, real-world example	CRASH-2, Traumabase registry
Park et al. [72]	2024	A step-by-step approach for transportability	Synthesizing steps to address transportability problems in practice	Exchangeability, positivity	Real-world example	Working from home (WHF) trial, American time use survey (ATUS)
Rott et al. [73]	2024	Causal meta-analysis	Considering multiple adjustments to causal meta-analysis to stabilize estimators when assumptions are not fully met	Exchangeability, positivity	Real-world example	Real-time Continuous Glucose Monitoring Data, data on various chiropractic procedures
Scelo et al. [74]	2024	Observational transportability	Primer article to observational transportability	Exchangeability, positivity	Simulation, real-world example	NINFEA study, Piedmont region birth register
Wang et al. [75]	2020	Treatment importance metric	Establishing a global treatment importance metric for comparability between different drug regimen in the same target population	Exchangeability, positivity	Simulation, real-world example	31 international observational studies on treatment of multidrug-resistant tuberculosis
Webster-Clark et al. [64]	2024	Transportability methods in distributed network studies	Showing how transportability might be applied and the potential value of transportability methods in distributed network studies	Exchangeability, positivity	Simulation	–
Webster-Clark et al. [76]	2024	Covariate selection	How inclusion of non-effect measure modifiers affects the estimation of the transported risk difference	Exchangeability, positivity	Simulation	–
Webster-Clark & Keil [77]	2023	Choosing the minimal sufficient set given effect measure in transportability studies	Recommendations which covariates to include within transportability applications depending on effect measure	Common outcome model	Simulation	–
Westreich et al. [1]	2019	Arguments for addressing internal and external validity at the same time	Addressing internal and external validity with a common approach	Positivity, external interference equivalence	Toy examples	–
Zivich et al. [79]	2024	Transportability analysis with positivity violation	Presenting methods to address positivity violations using g-computation and inverse-odds estimators with a binary covariate	Exchangeability, positivity	Simulation, real-world example	GetTested data
Zivich et al. [78]	2024	Transportability analysis with positivity violation	Combining statistical and mathematical models to address positivity violations with a continuous covariate	Exchangeability	Simulation, real-world example	ACTG trials, Women’s Interagency HIV study

IOSW = inverse odds of sampling weights, MCAR = missing completely at random, MAR = missing at random

#### Methods introduced and problems addressed

The introduced transportability estimators for the risk difference with randomized data in the source population were the inverse odds of sampling weighting [24], outcome modelling [43], and the doubly robust estimators targeted maximum likelihood estimation [45], augmented inverse weighting [15] and entropy balancing [44]. Specific applications of transportability methods were observational data from the source population [9, 51], inference with relative effect measures [48], meta-analysis [13, 14, 52, 56, 60], mediation analysis [57, 58], survival analysis [47, 53], bridged treatment comparison [59, 63], usage of register data to debias selective trials [46], multicenter trials [54], subgroup analyses [55, 62], benchmarking observational results with RCT data from an external population [49], flexible treatment trials [61] and a target population with non-probability samples [50]. Studies of other methodological aspects addressed covariate selection [70, 76, 77], missing data [65, 71], violations of the positivity assumption [78, 79], sensitivity analysis [68, 69], stabilizing estimators in causal meta-analysis [73], bias and variance equations [67], a treatment importance metric [75], target validity [1] general modified approaches, reviews and primer articles [18, 64, 66, 72, 74].

#### Assumptions related to methods

Methodological studies mostly reported the assumptions of positivity (40/44) [1, 9, 13, 14, 18, 23, 24, 44–76] and exchangeability (39/44) [9, 13, 14, 18, 23, 24, 44–57, 59–65, 67–76, 78, 79]. Some studies explicitly state the assumption of a common outcome model (3/44) [45, 58, 77]. Other reported assumptions were method specific, like conditional linearity of potential outcomes and conditional log-odds of study participation (and treatment for an observational source population) [9, 44]. Further reported assumptions included no mediator outcome confounding in mediation analysis [57] and parametric assumptions about missingness [65, 71], the causal structure [66], and data missing at random [60].

#### Robustness of estimators for randomized data

Studies introducing augmented inverse weighting [23], entropy balancing [44] and targeted maximum likelihood estimation [45] for a transportability analysis stated doubly robustness of the estimators. Double robustness implies that the estimation is robust against misspecification of the outcome or sampling model. Robustness is reported for the outcome modelling approach when the outcome model is correctly specified [43] and for inverse odds of sampling weighting when the sampling model is correctly specified [24].

#### Type of transportability application

Methodological studies presented different types of transportability applications to illustrate the use of the newly introduced method. The most frequent approach was to use simulated and real-world examples in the same study (23/44) [9, 14, 18, 23, 44, 45, 47, 50, 53, 56–61, 66–68, 71, 74, 75, 78, 79]. Other studies used either real-world examples (13/44) [13, 43, 46, 48, 49, 52, 54, 55, 63, 69, 70, 72, 73] or only simulated examples (5/44) [51, 64, 65, 76, 77]. Some studies used toy examples in the form of a fictional but not simulated numerical application (3/44).

### Data sources used repeatedly in studies applying transportability methods

Several data sources were repeatedly used within and across study types. Data from the AIDS clinical trials group database (ACTG) were used most often in included studies (6/44) [33, 53, 59, 63, 69, 79]. MTO trial data (5/44) [80] were used in transportability applications to exemplify new methods [45, 57, 58] and to analyze site differences [37, 38]. ACCORD-BP trial data (5/44) [81] were used to illustrate an estimator for randomized data [44], an estimator for the transportability of survival functions [47], to assess effect heterogeneity [27, 30] and to transport an estimated effect to a distinct target population [28]. Data from the CRASH trials in combination with Traumabase registry data were used in several studies with a methodological focus (4/44) [18, 67, 68, 71]. SPRINT trial data (2/45) [82] were reused as the source population data in multiple applied studies [30, 31]. HALT-C trial data (2/45) [83] were used in two studies of transportability methods in a meta-analysis [13, 14]. Data from the MOSAIC trial [32, 70, 84] and the CASS trial [23, 48, 85] were used twice, respectively. As a target population, the NHANES survey (3/44) [28, 30, 44] and Medicaid data (2/44) [39, 40] were used multiple times. Other data sources were used once within the reviewed studies (Tables 1 and 3).

## Discussion

### Summary of findings

We conducted a scoping review of 20 applied studies of transportability methods and 44 methodological studies with a transportability application. Applied studies used transportability methods with different constellations of source and target population data. Most applied studies used multicenter RCTs for source population data. Studies without complete treatment and outcome data on the target population commonly aimed to transport an estimated effect to a larger population of interest. Studies with complete treatment and outcome data across populations commonly aimed to assess whether effect heterogeneity between populations can be explained by measured effect modifiers or mediators.

### Challenges and outlook

The rising number of studies with applications of transportability methods found by this scoping review is in line with expectations that transportability methods are becoming increasingly important for causal inference research [86]. However, only few studies applied transportability methods to practical research questions. Variability in how transportability methods were applied together with sparse and inconsistent guidance on their application may currently hinder their broader adoption. Covariate selection is a crucial decision in avoiding bias and lowering the variance of transported estimates [7, 67, 76]. We found different approaches (and lack of a reported approach) to covariate selection in applied studies. This is in line with differing suggestions from the literature for covariate selection, such as focusing on potential effect modifiers [19, 23, 87] or effect modifiers differing between the source and target populations [16, 88], by considering the causal structure underlying an effect [6, 7] or by applying a selection algorithm [89]. Another central decision in transportability applications is the choice of the transportability estimator. Most applied studies used inverse odds of sampling weights or outcome modelling as a transportability estimator, despite the availability of doubly robust estimators. More guidance on choosing and implementing transportability estimators for specific applications could promote the use of transportability methods. For instance, outcome modelling could be a better choice than weighting approaches for small target population samples [43]. Future research could further explore applications of transportability methods to transporting effects from quasi-experimental study designs, such as difference-in-difference [90] or regression discontinuity [91] studies.

### Strengths and limitations

The strengths of this scoping review include a literature search in several databases and an iterative approach to data extraction from included studies, during which we refined the data extraction while reviewing studies. A limitation of this review is that studies of generalizability applications, in which the source and target populations fully overlap, were not considered. Other limitations are the exclusion of studies with a non-numerical application during the screening and the exclusion of grey literature and studies published after December 18, 2024, or before 2010 from the literature search. Aligned with the aims of this review to scope the literature, we did not assess the quality of reviewed studies.

## Conclusions

By describing why transportability methods were used, when transportability methods were applied and how transportability methods were implemented in applied studies, we mapped and characterized applications of transportability methods. More guidance and recommendations on when, why and how to transport estimated effects could provide a useful orientation for applying transportability methods. More applications of transportability methods to practical research questions could promote a broader adoption of this approach to improve the external validity of research results when a target population differs from a study population.

## Electronic supplementary material

Below is the link to the electronic supplementary material.

Supplementary Material 1

Supplementary Material 2
