# Supplementary material for: When, why and how are estimated effects transported between populations? A scoping review of studies applying transportability methods
Source: Eur J Epidemiol. 2025 Apr 18;40(3):255–73. doi: 10.1007/s10654-025-01217-w (PMC12137380; doi:10.1007/s10654-025-01217-w)
Supplement: Supplementary file 2 — Supplementary Material 2 [file 10654_2025_1217_MOESM2_ESM.docx]

**Supplement B:** Search strategy used to identify studies applying transportability methods

**MEDLINE (Ovid)**

(

Transportability.ti,ab,kf.

**OR** Transporting.ti,ab,kf.

)

**AND**

(

"Treatment Outcome"/

**OR**

(

"Causal Effect*"

**OR** "Treatment Effect*"

**OR** "Intervention Effect*"

**OR** Estimate*

**OR**

(

(

Experimental

**OR** RCT*

**OR** trial*

**OR** stud*

**OR** research

) ADJ3 Result*

)

**OR** Outcome*

).ti,ab,kf.

)

**AND** 2010:2025.(yr).

**NOT** Transporter*.ti,ab,kf.

Result on December 18, 2024: 1353 articles

**Embase**

(

Transportability:ti,ab,kw

**OR** Transporting:ti,ab,kw

)

**AND**

(

"Treatment Outcome"/de

**OR**

(

"Causal Effect*"

**OR** "Treatment Effect*"

**OR** "Intervention Effect*"

**OR** Estimate*

**OR**

(

(

Experimental

**OR** RCT*

**OR** trial*

**OR** stud*

**OR** research

) NEAR/3 Result*

)

**OR** Outcome*

):ti,ab,kw

)

**AND** [2010-2025]/py

**NOT** Transporter*:ti,ab,kw

**NOT** "conference abstract"/it

Result on December 18, 2024: 1318 articles

**Web of Science**

TS=(

(

Transportability

**OR** Transporting

)

**AND**

(

"Causal Effect*"

**OR** "Treatment Effect*"

**OR** "Intervention Effect*"

**OR** Estimate*

**OR**

(

(

Experimental

**OR** RCT*

**OR** trial*

**OR** stud*

**OR** research

) NEAR/2 Result*

)

**OR** Outcome*

)

**NOT** Transporter*

)

**AND** PY=(2010-2025)

Result on December 18, 2024: 2960 articles

**EconLit (EBSCO)**

(

Transportability

**OR** Transporting

)

**AND**

(

"Causal Effect*"

**OR** "Treatment Effect*"

**OR** "Intervention Effect*"

**OR** Estimate*

**OR**

(

(

Experimental

**OR** RCT*

**OR** trial*

**OR** stud*

**OR** research

) N2 Result*

)

**OR** Outcome*

)

**NOT** Transporter*

**AND** DT 2010-2025

Result on December 18, 2024: 61

**Google Scholar**

Transportability

**OR** Transporting "Causal Effect"

**OR** "Treatment Effect"

**OR** "Intervention Effect"

**OR** Estimate

**OR**

"

Experimental

**OR** RCT

**OR** trial

**OR** study

**OR** research AROUND(3) Result

"

**OR** Outcome

**Years:** 2010–2025

Result on December 18, 2024: Top 100

**Inclusion criteria**

1. A numerical analysis was conducted in which an average treatment effect or potential outcome means were transported from a source to a target population.
2. The analysis needed to target a setting in which at least some members of the source population sample have a zero percent probability of being in the sample from the target population.

**Exclusion criteria**

None
